# Risk Factors Associated with Mortality in Severe Chest Trauma Patients Admitted to the ICU

**DOI:** 10.3390/jcm11010266

**Published:** 2022-01-05

**Authors:** Jesús Abelardo Barea-Mendoza, Mario Chico-Fernández, Manuel Quintana-Díaz, Jon Pérez-Bárcena, Luís Serviá-Goixart, Ismael Molina-Díaz, María Bringas-Bollada, Antonio Luis Ruiz-Aguilar, María Ángeles Ballesteros-Sanz, Juan Antonio Llompart-Pou, on behalf of the Neurointensive Care and Trauma Working Group of the Sociedad Española de Medicina Intensiva Crítica y Unidades Coronarias (SEMICYUC)

**Affiliations:** 1 UCI de Trauma y Emergencias, Servicio de Medicina Intensiva, Hospital Universitario 12 de Octubre, 28041 Madrid, Spain; elbarea@gmail.com (J.A.B.-M.); murgchico@yahoo.es (M.C.-F.); 2 Servicio de Medicina Intensiva, Hospital Universitario La Paz, 28046 Madrid, Spain; mquintanadiaz@gmail.com; 3 Servei de Medicina Intensiva, Hospital Universitari Son Espases, Institut d’Investigació Sanitària Illes Balears (IdISBa), 07120 Palma de Mallorca, Spain; juan.perez@ssib.es; 4 Servei de Medicina Intensiva, Hospital Universitari Arnau de Vilanova, Universitat de Lleida, IRBLleida, 25198 Lleida, Spain; lserviag@gmail.com; 5 Servicio de Medicina Intensiva, Hospital Universitario Nuestra Señora de la Candelaria, 38010 Santa Cruz de Tenerife, Spain; imolinad@hotmail.com; 6 Servicio de Medicina Intensiva, Hospital Clínico Universitario San Carlos, 28040 Madrid, Spain; brinsk@hotmail.com; 7 Servicio de Medicina Intensiva, Hospital Universitario Miguel Servet, 50009 Zaragoza, Spain; alruiz@salud.aragon.es; 8 Servicio de Medicina Intensiva, Hospital Universitario Marqués de Valdecilla, 39008 Santander, Spain; gelesballesteros@yahoo.com

**Keywords:** chest trauma, thoracic trauma, severe trauma, intensive care, risk factors, RETRAUCI

## Abstract

Our objective was to determine outcomes of severe chest trauma admitted to the ICU and the risk factors associated with mortality. An observational, prospective, and multicenter registry of trauma patients admitted to the participating ICUs (March 2015–December 2019) was utilized to collect the patient data that were analyzed. Severe chest trauma was defined as an Abbreviated Injury Scale (AIS) value of ≥3 in the thoracic area. Logistic regression analysis was used to evaluate the contribution of severe chest trauma to crude and adjusted ORs for mortality and to analyze the risk factors associated with mortality. Overall, 3821 patients (39%) presented severe chest trauma. The sample’s characteristics were as follows: a mean age of 49.88 (19.21) years, male (77.6%), blunt trauma (93.9%), a mean ISS of 19.9 (11.6). Crude and adjusted (for age and ISS) ORs for mortality in severe chest trauma were 0.78 (0.68–0.89) and 0.43 (0.37–0.50) (*p* < 0.001), respectively. In-hospital mortality in the severe chest trauma patients without significant traumatic brain injury (TBI) was 5.63% and was 25.71% with associated significant TBI (*p* < 0.001). Age, the severity of injury (NISS and AIS-head), hemodynamic instability, prehospital intubation, acute kidney injury, and multiorgan failure were risk factors associated with mortality. The contribution of severe chest injury to the mortality of trauma patients admitted to the ICU was very low. Risk factors associated with mortality were identified.

## 1. Introduction

Severe trauma remains a major public health problem. It constitutes the leading cause of death, hospitalization, and long-term disabilities in young patients [1]. Trauma patients admitted to the intensive care unit (ICU) usually present injuries in different anatomical areas [2]. In our environment, chest trauma constitutes the second most severely affected anatomical area among trauma ICU patients [2].

Whilst consensus exists regarding the important role of severe chest trauma in prehospital mortality in patients presenting with hemodynamic instability [3,4], its exact contribution to the mortality rates of trauma patients admitted to the ICU and its associated risk factors remain to be determined. Some authors suggest that it directly accounts for approximately 25% of trauma related-mortality and is a contributing factor in another 25% of cases [5], remaining unchanged over the years [6], whilst others suggest that its contribution to mortality is almost irrelevant [7,8]. The association of extra-thoracic injuries is common in this population [8].

Due to this controversy, our objective in this multicenter study was to determine outcomes of severe chest trauma patients admitted to the ICU and the risk factors associated with mortality, using data from the Spanish Trauma ICU Registry (RETRAUCI).

## 2. Materials and Methods

The RETRAUCI is an observational, prospective, and multicenter nationwide registry that currently includes 52 ICUs in Spain. It is endorsed by the Neurointensive Care and Trauma Working Group of the Spanish Society of Intensive Care Medicine (SEMICYUC) and currently operates in a web-based electronic system (www.retrauci.org, accessed on 10 December 2021). Ethics Committee approval for the registry was obtained (Hospital Universitario 12 de Octubre, Madrid: 12/209). Informed consent was not obtained for this specific study, since this was a retrospective analysis of de-identified collected data.

We included in this study all adult patients admitted to the participating ICUs between March 2015 and December 2019. Patients were managed according to the Advanced Trauma Life Support principles. In this population, we analyzed the incidence, outcomes, and risk factors associated with mortality in severe chest trauma patients. Data on the epidemiology, acute management in the pre-hospital and in-hospital stages, type and severity of injury, resource utilization, complications, and outcomes were recorded. We only excluded patients with incomplete data or unknown hospital outcomes.

### 2.1. Definitions

- Severe chest trauma was defined as an Abbreviated Injury Scale (AIS) value of ≥3 in the thoracic area. The control group included patients without chest trauma and those with thoracic AIS ≤ 2 [8].
- Hemodynamic condition was considered as follows [9]:
  - Stable, systolic blood pressure > 90 mmHg during initial trauma care.
  - Unstable, responding to volume replacement—systolic blood pressure < 90 mmHg requiring volume replacement for normalization.
  - Shock, systolic blood pressure < 90 mmHg requiring volume replacement, blood products, and vasoactive support for normalization.
  - Refractory shock, hypotension refractory to volume replacement, blood products, or vasoactive support and activation of the massive bleeding protocol.
- Rhabdomyolysis, laboratory test determination of creatine kinase > 5000 U/L [10]^.^
- Acute kidney injury (AKI) was evaluated by using the Risk, Injury, Failure, Loss of kidney function and End-stage kidney disease (RIFLE) definition [10].
- Trauma-associated coagulopathy, prolongation of the prothrombin and activated partial thromboplastin over 1.5 times the control values, fibrinogen < 150 mg/dL, or thrombocytopenia (<100,000/µL) in the first 24 h [9].
- Multiorgan failure was defined, using the Sequential-related Organ Failure Assessment (SOFA), as the alteration of two or more organs with a score of ≥3 [9].
- Massive hemorrhage was defined as the need for more than 10 packed red blood cell concentrates in the initial 24 h.

### 2.2. Statistical Analysis

Quantitative variables are shown as means ± standard deviations (SDs) and qualitative variables as numbers (percentages). Categorical variables were analyzed using the χ^2^ or Fisher’s exact test. For continuous data, we studied normality with the Shapiro–Wilk test. Continuous data were evaluated using the Student’s t-test or the non-parametric Kruskal–Wallis test in the case of a non-normal distribution. We analyzed the contribution of severe chest trauma to crude and adjusted (for age and ISS) ORs for mortality by using logistic regression analyses. A multiple logistic regression analysis was performed to analyze the risk factors associated with death in severe chest trauma patients. The variables entered in the logistic regression analysis were those significantly associated with death in the univariate analysis. A *p*-value of <0.10 was considered significant. Results are presented as odds ratios (ORs) with 95 percent confidence intervals (95% CI). The calibration and goodness-of-fit of the logistic regression models were evaluated using the χ^2^ Hosmer–Lemeshow (HL) test, and model discrimination was assessed by means of the area under the receiver operating characteristic curve (AUROC) analysis. We reported all results as stated in the RECORD statement [11]. Statistical analysis was performed with STATA 15 (StataCorp. 2017).

## 3. Results

During the study period, 9790 trauma patients were admitted to the participating ICUs. The mean age of the sample was 49.88 (19.21) years, 77.6% were male, 93.9% had presented trauma, the mean ISS score was 19.9 (11.6), and the mean NISS score was 25.63 (14.62). The distribution of the severity of thoracic injury according to the AIS was: 4752 patients (48.54%) with no thoracic involvement, 183 patients (1.87%) with thoracic AIS 1, 1034 patients (10.56%) with thoracic AIS 2, 2294 patients (23.43%) with thoracic AIS 3, 1060 patients (10.83%) with thoracic AIS 4, 458 patients (4.68%) with thoracic AIS 5, and 9 patients (0.09%) with thoracic AIS 6. Therefore, up to 3821 patients (39%) presented with a thoracic AIS ≥ 3; these patients constituted the study population and the remaining 5969 comprised the control group.

Baseline characteristics of the population with severe chest trauma and the control group are summarized in Table 1.

The percentage of patients with AIS values of ≥3 in the different areas in the severe chest trauma and the control groups are shown in Table 2. The mean values (SD) of the AIS values in the different areas are summarized in Table 3.

Urgent (<24 h) cardiothoracic (4.34% vs. 0.37%, *p* < 0.001) and abdominal (8.35% vs. 4.72%, *p* < 0.001) surgeries were more frequent in the severe chest trauma group, whereas urgent (<24 h) neurosurgical procedures were more frequent in the control group (16.32% vs. 5.78%, *p* < 0.001). In the initial 24 h, 30.46% of patients with severe chest trauma received packed red blood cell concentrates, and 22.86% received fresh frozen plasma. In the control group, 19.62% of patients received packed red blood cell concentrates, and 14.48% received fresh frozen plasma (both *p*s < 0.001). Bleeding control angiography was used more often in the severe chest trauma group (7.40% vs. 5.83%, *p* = 0.004), as well as the need for tracheostomy (13.17% vs. 10.18%, *p* < 0.001).

Patients with severe chest trauma presented a higher percentage of complications because of the higher severity of injury (Table 3). Intracranial hypertension was more frequent in the control group since the severity of brain injury, as measured by the AIS-head, was higher (Table 4).

**Figure 1:**
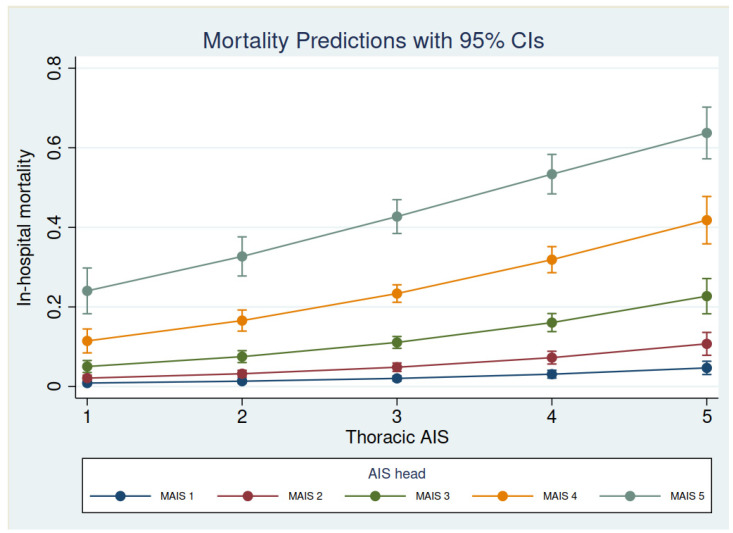
In-hospital mortality prediction in chest trauma according to the severity of head injury.

Mechanical ventilation was more frequently used in the control group, likely because of the higher incidence of severe head injury; however, the length of mechanical ventilation and the ICU length of stay were higher in the severe chest trauma group (Table 5). Despite the higher severity of injury, both ICU and in-hospital mortality were lower in the severe chest trauma group. Crude and adjusted (for age and ISS) ORs for mortality in the severe chest trauma group were 0.78 (0.68–0.89) and 0.43 (0.37–0.50), *p* < 0.001, respectively. Of note, up to 34% of the deceased in the severe trauma group died because of intracranial hypertension (Table 4). As a result, we explored the mortality in the severe chest trauma group according to the coexistence of severe head injury (AIS-head ≥ 3). In-hospital mortality in the severe chest trauma group without significant traumatic brain injury (TBI) was 5.63% and was 25.71% with associated significant TBI, *p* < 0.001. The relationship between chest and brain injury and in-hospital mortality is shown in Figure 1.

Multiple logistic regression analyses were performed to analyze the risk factors associated with mortality in the severe chest trauma group. Age, the severity of injury evaluated by the NISS and the AIS-head, hemodynamic instability, the need for prehospital intubation, and the development of acute kidney injury and multiorgan failure were independently associated with mortality. On the other hand, nosocomial infection, trauma-associated coagulopathy, and the need for tracheostomy were protective factors (Table 6).

**Figure 2:**
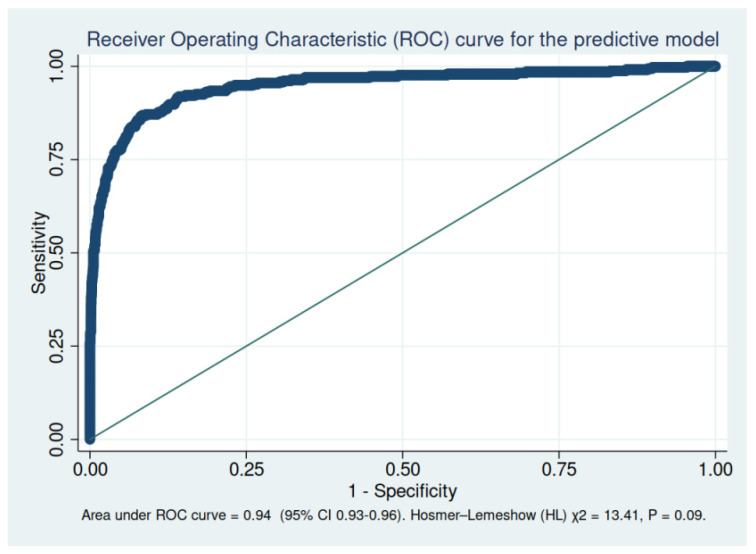
Receiver operating characteristic (ROC) curve for the predictive model.

The values in parentheses represent the 95 percent confidence intervals. Variables with *p* < 0.10 in univariate analysis were entered into the multivariable models. The area under the receiver operating characteristic curve (AUROC) was 0.94 (95% CI, 0.93–0.96). The result of the Hosmer–Lemeshow (HL) test was χ^2^ = 13.41, *p* = 0.09. (Figure 2)

## 4. Discussion

The main finding of our study was that severe chest trauma was associated with a low mortality burden once the patient is admitted to the ICU. In addition, we identified different risk factors associated with mortality, including age, the severity of the injury, brain injury, hemodynamic instability, the need for prehospital intubation, and the development of acute kidney injury and multiorgan failure.

The contribution of chest trauma to the mortality of trauma patients remains controversial since studies have shown contradictory results. Single-center studies have shown a low mortality rate even considering higher ISS values [7,8], but data obtained from multicenter registries show a higher mortality rate [6,12] that has remained stable over the last few years [6]. We observed a low mortality rate in patients with severe chest injury admitted to the ICU, even considering the higher ISS in this population, as shown by Grubmüller et al. [8]. Moreover, mortality was highly dependent on the severity of brain injury, as determined by the AIS-head value. In-hospital mortality in severe chest trauma with AIS-head values <3 was only 5.63%. The interaction of brain injury and chest trauma in the outcomes of trauma patients has been described elsewhere in an inverse manner [13,14]. In our series, the probability of mortality with AIS-head 1–3 was very low, even in cases with thoracic AIS 4 and 5, thus indicating a low burden of mortality associated with the thoracic injuries.

Although our study was not designed for this purpose, the low mortality rates that were found were likely secondary to the use of a standardized approach, including local and systemic analgesia, a chest drain when necessary, non-invasive ventilation, lung-protective ventilation if acute respiratory distress syndrome is developing, early extubation, the use of extracorporeal membrane oxygenation, and/or rib fixation [15,16,17,18].

We additionally identified risk factors associated with mortality in chest trauma patients, including age, the severity of the trauma, and the severity of brain injury; variables related to physiological trauma, such as hemodynamic instability and the need for prehospital intubation; and the development of complications, such as acute kidney injury and multiorgan failure. Some factors were associated with a lower mortality, likely reflecting a longer ICU length of stay due to the lower mortality rates found (nosocomial infection and need of tracheostomy), rather than a protective role. The control group had a higher incidence of severe brain injury and the development of intracranial hypertension, which was a major determinant of death (57.26% of cases in the control group).

Previous studies have identified factors associated with mortality in chest trauma. Huber et al. identified severe vessel intrathoracic injuries, bilateral lung contusions, bilateral major lacerations, bilateral flail chest, age, blood transfusion, initial and admission hypotension (<90 mmHg), AIS-head ≥ 3, AIS-abdomen ≥ 3, and structural heart injury (AIS ≥ 3) as the risk factors associated with mortality [19]. In a single-center study, Söderlund et al. identified the degree of hypoperfusion (base excess) and coagulation abnormalities (thromboplastin time) at admission as risk factors associated with mortality [20]. In a systematic review, Battle et al. found that age, the number of rib fractures, the presence of pre-existing disease, and pneumonia to be related to mortality in 29 identified studies [21].

The main strength of our study is the large sample of trauma patients admitted to the participating ICUs. To date, this is the largest sample evaluated in Spain, and we believe this study clearly delineates the epidemiology and outcomes of severe chest injury in our environment. This supports the usefulness of trauma registries in the management and benchmarking of severe trauma patients [22,23].

However, limitations must be acknowledged too. Despite patients being managed following the Advanced Trauma Life Support principles, we cannot rule out deviations, so this could affect patients’ management and outcomes. In addition, the inclusion criteria of being admitted to the participating ICUs may not reflect the critical trauma population due to differences in the admission criteria and bed and staffing availability. Lastly, Trauma-associated coagulopathy was associated with a protective effect in terms of the mortality of severe chest trauma patients. Trauma-associated coagulopathy is usually associated with a higher burden of morbidity and mortality [4,24]. We were unable to identify the underlying reason for this observed effect. We acknowledge that a detailed analysis of the different types of thoracic injuries—differentiating vascular, cardiac, airway disruptions injuries—might improve the comprehension of our results.

## 5. Conclusions

In conclusion, we found that the contribution of severe chest injury to the mortality of trauma patients admitted to the ICU is very low. Risk factors associated with mortality included age, the severity of the trauma, the severity of brain injury, hemodynamic instability, the need for prehospital intubation, and the development of acute kidney injury and multiorgan failure.

## Figures and Tables

**Table 1:** Baseline characteristics of the population with severe chest trauma and the control group.

	Severe Chest Trauma *n* = 3821	Control Group *n* = 5969	*p*-Value
Age	49.72 (18.28)	49.97 (19.79)	0.668
Sex	79.53%	76.47%	<0.01
Penetrating	5.26%	6.50%	0.012
ISS	25.60 (12.39)	16.12 (9.47)	<0.001
ISS ≥16	80.53%	53.68%	<0.01
NISS	31.27 (13.75)	22.02 (14.00)	<0.001
Mechanism			<0.01
Ground-level fall	11.70%	30.41%	
RTA-car	21.49%	13.49%	
Precipitation	18.42%	11.83%	
RTA-motorcycle	20.70%	12.95%	
RTA-run over	8.40%	8.56%	
Other	19.29%	23.67%	
Prehospital mobile ICU	74.33%	70.95%	<0.01
Prehospital intubation	22.03%	22.41%	0.825
Hemodinamically stable	56.71%	70.02%	<0.01

ISS, injury severity score; NISS, new injury severity score; RTA, road traffic accident; ICU, intensive care unit.

**Table 2:** Percentage of patients with AIS ≥ 3 in the different areas in the severe chest trauma and control groups.

	Severe Chest Trauma *n* = 3821	Control Group *n* = 5969	*p*-Value
Head	30.83%	55.79%	<0.001
Face	2.88%	3.17%	0.420
Abdomen	18.35%	12.00%	<0.001
Extremities	22.27%	17.62%	<0.001
External	0.24%	1.71%	<0.001

**Table 3:** Mean values (SD) of the AIS values in the different areas in the severe chest trauma and control groups.

	Severe Chest Trauma *n* = 3821	Control Group *n* = 5969	*p*-Value
Head	3.06 (1.23)	3.56 (1.15)	<0.001
Face	1.79 (0.73)	1.79 (0.73)	0.896
Abdomen	2.68 (0.94)	2.80 (0.90)	<0.001
Extremities	2.52 (0.90)	2.61 (0.95)	<0.001
External	1.25 (1.56)	2.26 (1.56)	<0.001

**Table 4:** Percentage of complications in the severe chest trauma and control groups.

	Severe Chest Trauma *n* = 3821	Control Group *n* = 5969	*p*-Value
Rhabdomyolysis	22.46%	11.33%	<0.001
Trauma-associated coagulopathy	20.14%	13.41%	<0.001
Massive hemorrhage	9.20%	4.21%	<0.001
Acute kidney injury	22.53%	13.88%	<0.001
Intracranial hypertension	11.44%	20.37%	<0.001
Respiratory failure (PaO_2_/FiO_2_ < 300)	39.94%	17.77%	<0.001
Nosocomial infection	23.34%	19.97%	<0.001
Multiorgan failure	14.76%	7.01%	<0.001

**Table 5:** Main outcomes in the severe chest trauma and control groups.

	Severe Chest Trauma *n* = 3821	Control Group *n* = 5969	*p*-Value
Angioembolization	7.4%	5.83%	0.004
MV	45.09%	50.96%	<0.001
Days of MV (if ≥1 day)	10.58 (12.50)	7.46 (11.00)	<0.001
ICU LOS	9.97 (16.33)	7.85 (12.48)	<0.001
ICU mortality	10.43%	12.95%	<0.001
In-hospital mortality	11.81%	15.00%	<0.001
Cause of death			<0.001
Exsanguination	13.56%	4.09%	
Intracranial hypertension	34.84%	57.26%	
Multiorgan failure	30.05%	14.39%	
Other	21.54%	24.26%	

MV, mechanical ventilation; ICU, intensive care unit.

**Table 6:** Risk factors associated with mortality in severe chest trauma using multiple logistic regression analyses.

Variable	OR (95% CI)	*p*-Value
Age	1.03 (1.02–1.04)	<0.001
NISS	1.02 (1.01–1.04)	<0.001
AIS-head		
AIS-head 2	1.92 (1.03–3.58)	0.039
AIS-head 3	1.88 (1.06–3.34)	0.030
AIS-head 4	5.84 (3.29–10.36)	<0.001
AIS-head 5	15.92 (8.66–29.26)	<0.001
Hemodynamics		
Unstable volume-response	1.91 (1.01–3.59)	0.044
Shock	4.70 (2.89–7.65)	<0.001
Refractory shock	73.52 (37.73–143.27)	<0.001
Prehospital intubation	2.18 (1.55–3.05)	<0.001
Multiorgan failure	2.82 (1.82–4.38)	<0.001
Acute kidney injury	1.89 (1.27–2.81)	0.001
Nosocomial infection	0.41 (0.26–0.62)	<0.001
Trauma-associated coagulopathy	0.87 (0.79–0.96)	0.006
Tracheostomy	0.08 (0.04–0.15)	<0.001

NISS, new injury severity score; AIS, abbreviated injury scale.

## Data Availability

Data can be obtained from the authors upon reasonable request.
